# Insights into the Isolation, Identification, and Biological Characterization Analysis of and Novel Control Strategies for *Diaporthe passiflorae* in Postharvest Passion Fruit

**DOI:** 10.3390/jof9101034

**Published:** 2023-10-20

**Authors:** Huiling Wang, Hongbin Chen, Yu Lin, Meiling Li, Qingqing Liu, Yuzhao Lin, Xuanjing Jiang, Yihui Chen

**Affiliations:** 1 College of Oceanology and Food Science, Quanzhou Normal University, Quanzhou 362000, China; 2 Institute of Postharvest Technology of Agricultural Products, College of Food Science, Fujian Agriculture and Forestry University, Fuzhou 350002, China; 3 Department of Intelligent Manufacturing, MinXi Vocational and Technical College, Longyan 364021, China

**Keywords:** *Passiflora caerulea*, postharvest diseases, *Diaporthe passiflorae*, biological characteristics, antifungal

## Abstract

Postharvest diseases seriously restrict developments in the passion fruit industry. In this study, we aimed to identify the postharvest pathogen affecting passion fruit, investigate its pathogenicity, and explore relevant control methods. The pathogen was isolated from rotting passion fruit and identified using morphological characteristics, ITS sequences, and phylogenetic tree analyses. Additionally, preliminary studies were conducted to assess the biological characteristics of the pathogen and evaluate the efficacy of various treatments for disease control. The fungus on the passion fruit called B4 was identified as *Diaporthe passiflorae*. Optimal conditions for mycelial growth were observed at 25–30 °C and pH 5–6, with starch as the carbon source and peptone as the nitrogen source. Infection by *D. passiflorae* accelerated fruit decay, reduced the *h°* value of the peel, and increased the peel cell membrane permeability when compared to the control. Notably, treatments with appropriate concentrations of ɛ-poly-l-lysine, salicylic acid, and melatonin showed inhibitory effects on the pathogen’s growth in vitro and may thus be potential postharvest treatments for controlling brown rot caused by *D. passiflorae* in passion fruit. The results provide a scientific basis for the development of strategies to control postharvest decay and extend the storage period of passion fruit.

## 1. Introduction

Passion fruit (*Passiflora cerulean* L.) is a perennial, evergreen, climbing woody vine that originates from South America but is now widely cultivated in tropical and subtropical regions worldwide [1]. Brazil is the world’s largest producer of passion fruit, with an annual production of 683,000 tons in 2021, covering an area of about 45,000 hectares and generating an income of about USD 286 million for farmers [2]. In China, passion fruit is mainly grown in the provinces of Fujian, Hainan, Guangdong, and Guangxi [3]. In 2019, the cultivation area of passion fruit in China was about 30,000 hectares, and the annual production was about 600,000 tons [4]. Passion fruit produces a unique aroma for a tropical fruit, as it contains the scents of apple, mango, pineapple, banana, lemon, and litchi fruit. Its juice is sweet and sour, with a unique flavor, and it is known colloquially as the “king of fruit juices” [5,6,7]. Passion fruit is also rich in various phenolic compounds, dietary fiber, pectin, carotene, vitamins, and other active substances required by the human body, and it has antioxidant, antihypertensive, antitumor, hypolipidemic, and other medicinal values [8,9,10]. Owing to its unique flavor and high nutritional value, passion fruit is sought after by consumers, and the market demand for passion fruit is increasing, thereby leading to its high commercial value; thus, the planting area of passion fruit is increasing. For example, the planted area of passion fruit in China increased from less than 700 hectares in 2011 to more than 30,000 hectares in 2019, with an annual production of about 600,000 tons [4,11].

However, as passion fruit ripens during the high-temperature season and has a high level of respiratory action [12,13], the peel can easily lose its luster and experience water loss, shrinkage, and rotting, which makes storage challenging [14,15]. Additionally, passion fruit is susceptible to pathogen infections that lead to fruit rot. These problems seriously affect the quality and commercial value of passion fruit. Globally, several fungal pathogens have been identified as causing passion fruit decays, including *Diaporthe infercuda* [16], *Phytophthora drechsleri* [17], *Phytophthora nicotianae* [18], *Colletotrichum brevisporum* [19], *Lasiodiplodia theobromae* [20], and *Trichothecium roseum* [21].

To improve the shelf life of passion fruit and control the occurrence of postharvest diseases, chemical preservation treatments are currently widely used in the market [4,12,22]. However, the prolonged use of chemical fungicides can lead to a series of problems, including the development of pathogen resistance, the contamination of the environment, and harm to human health [23,24]. Therefore, it is necessary to find new safety strategies to control the occurrence of postharvest diseases. ɛ-poly-l-lysine (ε-PL) is a natural preservative with easy solubility and nontoxic, harmless, and antifungal characteristics, and it has been widely used in the food industry [25,26]. Both salicylic acid (SA) and melatonin (MT) treatments can delay fruit senescence and help maintain the postharvest quality [27,28,29,30]. Therefore, this study aimed to isolate and identify the pathogen causing brown rot in passion fruit, as well as its biological characteristics, and to explore the effects of ε-PL, SA, and MT treatments on the fungal pathogen in vitro.

## 2. Materials and Methods

### 2.1. Materials and Reagents

The ‘Fujian Passion Fruit No. 3’ passion fruits were collected from an orchard in Nan’an City, Fujian Province, China, and shipped to Quanzhou on the same day. Fruits of a uniform size, similar color, consistent maturity, no disease, no damage, and that were apparently healthy were selected for the experiment. A total of 390 passion fruits were selected after cleaning and subjected to the following treatments. Among them, 30 passion fruits were used for measuring the initial fruit indicators (day 0). The remaining 360 passion fruits were randomly divided into two groups, with 180 fruits in each group. One group was punched, and the other group was punched and placed with 8 mm *D. passiflorae* plugs. After treatment, 10 fruits were packed in polyethylene film bags (0.015 mm thick) and stored at 25 ± 1 °C and 85% relative humidity for 6 days. In the experiment, 30 passion fruits (3 bags) from each treatment were randomly selected every day for physiological index analysis.

### 2.2. Isolation and Purification of Pathogens

Postharvest passion fruits were packed in 0.015 mm polyethylene cling film bags and stored at 28 ± 1 °C and 85% relative humidity (RH) until the onset of decay. The margins (4 × 4 mm) between the symptomatic and healthy tissue were cut from the rotten fruit. Then, the surfaces were disinfected and cleaned with 75% ethanol [31]. The isolated tissue blocks were transferred to potato glucose agar (PDA) medium. Finally, the plates were cultured at constant temperature (28 °C) and humidity (85% RH) in an artificial climate chamber. When the colonies reached a diameter of approximately 3 cm, the mycelia at the edges of the colonies were placed into new PDA medium for purification, and this was repeated 3–4 times to obtain purified strains.

### 2.3. Morphological Identification of the Pathogen

The fungus plugs (Ø = 5 mm) were removed from the edge of the colony, inoculated on the PDA medium, and cultured in the artificial climate chamber with constant temperature (28 °C), humidity (85%), and darkness condition. The morphology of the pathogen colonies was observed, photographed, and recorded daily until the colonies covered the Petri dish on the fifth day. The prepared Sabouraud agar plate was cut into several squares (1 cm^2^) using a sterile inoculant needle via an aseptic procedure and placed on the agar plate [32,33]. The pathogens to be tested were inoculated on the upper part of the surrounding edge of the agar block, and then a sterile cover glass was placed on the agar with sterile tweezers and they were cultured at 28 °C under a plate lid. After the spores had grown on a sterile slide, a drop of normal saline was added, and the cultured cover slide was placed on the slide using sterile tweezers. Finally, the characteristics of the spores were observed and photographed with a light microscope (DM2000 LED, Leica, Wetzlar, Germany). Morphological characteristics were identified using relevant guidelines [34,35].

### 2.4. Pathogenicity Detection of the Pathogen

Pathogenicity testing was performed according to the method described by Chen et al. [31]. Healthy, disease-free, mature, and uniform-sized passion fruits were selected and washed with deionized water and dried naturally. The selected fruits were divided into three groups of 10 fruits each. A small hole (Ø = 8 mm) was punched on the equatorial surface of the passion fruit using a sterile puncher. After the wound was dried, the fungal plug (Ø = 8 mm) was inoculated, and the treatments without fungal plug inoculation were used as the controls. The fruits were then placed in an artificial climate box with constant conditions of 28 °C and 85% RH for preservation and photographed every day.

### 2.5. Molecular Biological Identification of Pathogens

DNA extraction, PCR amplification, and pathogen gene sequencing were performed by Qingdao Yixin Testing Technology Service (Qingdao, China). The ITS, TUB, and TEF-1α sequencing results of strain B4 were analyzed using the Basic Local Alignment Search Tool (BLAST) in the GenBank database. Subsequently, by comparing the similarity between the B4 strain sequence and the existing sequences in the database, the sequences with high similarity were obtained. Finally, DNAMAN software (Version 9.0, LynnonBiosoft, Chicago, IL, USA) was used for sequence comparison and homology analysis, and MEGA11.0 (Mega Limited, Auckland, New Zealand) was used for neighbor-joining analysis to construct the phylogenetic tree [36].

### 2.6. Biological Characteristics of the Pathogens

The biological properties were determined as described by Gui et al. [37] with slight modifications. The pathogen was inoculated on different carbon source (D-fructose, glucose, sucrose, and starch) and nitrogen source (peptone, KNO_3_, and NaNO_3_) media. The pathogens were also inoculated on PDA media with different pH values (4, 5, 6, 7, 8, 9, 10, and 11). The PDA medium inoculated with the pathogen was incubated in an artificial climate chamber at 85% RH and different temperatures (20 °C, 25 °C, 28 °C, 30 °C, and 35 °C). After 4 days, the diameters of the colonies on the medium were measured using the crossover method and then photographed and recorded.

### 2.7. In Vivo Assay

#### 2.7.1. Assay of Wound Inoculation with *D. passiflorae*

Passion fruits of uniform size that were healthy, disease-free, ripe, and consistent were selected. The samples were rinsed with deionized water and dried naturally. A small wound (Ø = 8 mm) was made in the passion fruit with an equatorial plane using a sterile puncher. After the wound had dried, a *D. passiflorae* cake with a diameter of 8 mm was inoculated [20]. Ten fruits were randomly selected from the treatment and control groups each day for observation. The diameters of the lesions were then measured using the crossover method and photographed for record.

#### 2.7.2. Effects of *D. passiflorae* Inoculation on the Fruit Hue Angle

The hue angle of the fruit was determined as described by Zhao et al. [38]. Ten fruits were randomly selected from the fruits inoculated with *D. passiflorae* and the control group each day. A colorimeter (CR400, Konica Minolta, Tokyo, Japan) was used to measure 4 points on the equatorial surface of the fruit. The *h°* value was recorded and averaged.

#### 2.7.3. Effects of *D. passiflorae* Inoculation on Cell Membrane Permeability

The determination of the cell membrane permeability for the fruit peel followed the method described by Shi et al. [39] with slight modifications. Thirty peels (5 mm diameter) from ten fruits were sampled and rinsed with distilled water. Then, the peels were transferred to a scale test tube containing 20 mL of distilled water. The peels were then allowed to stand for 1 h, and the initial electrolyte leakage (C1) was measured using a conductivity meter (STARTER3100C, Ohaus, Parsippany, NJ, USA). The scale test tube of the above extract was then boiled for 20 min and allowed to cool to measure the final electrolyte leakage (C2). The permeability of the fruit cell membranes was calculated according to the following formula:

Cell membrane permeability (%) = (C1/C2) × 100.

### 2.8. Effects of ε-PL, SA, and MT Treatments on the Mycelial Growth and Inhibition Rate of D. passiflorae In Vitro

In vitro inhibition experiments were carried out with slight modifications following the methods described by Fan et al. [40]. The ε-PL, SA, and MT were added to the PDA medium to give final concentrations of 0.125, 0.25, and 0.5 mg/mL of ε-PL, 0.4, 0.8, 1.2, and 1.6 mg/mL of SA, and 1, 2, 4, and 8 mg/mL of MT. Fungal plugs (Ø = 5 mm) were inoculated in the middle of the medium with different concentrations. The plates were incubated at a constant temperature (28 °C) and humidity (85% RH). Colony diameters were measured daily using the crossover method and then photographed and recorded.

### 2.9. Statistical Analysis

The described parameters were all tested in triplicate. The SPSS 22.0 software (IBM Corp, Armonk, NY, USA) was used to perform one-way ANOVA and *t*-tests on the experimental data. All data in the figures are expressed as average values ± standard error (n = 3). The differences between treatments are denoted by asterisks, indicating significant (* *p* < 0.05) or highly significant (** *p* < 0.01) differences. Microsoft Office Excel 2021 (Microsoft, Chicago, IL, USA) was used to produce all graphs.

## 3. Results

### 3.1. Symptoms of Brown Rot on Passion Fruit

**Figure 1:**
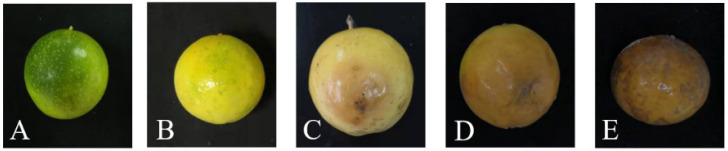
Symptoms of brown rot during postharvest storage of passion fruit: (**A**) 0 d; (**B**) 6 d; (**C**) 8 d; (**D**) 14 d; (**E**) 20 d.

The changes that occurred in the freshly picked healthy passion fruits that developed brown rot during postharvest storage are shown in Figure 1. The initial symptom of the disease is the appearance of water-stained spots (Figure 1C), and these develop in round pale-brown patches with expanding edges (Figure 1D). Ultimately, the color deepens and the fruit rots completely (Figure 1E).

### 3.2. Morphological Identification of Pathogens

**Figure 2:**
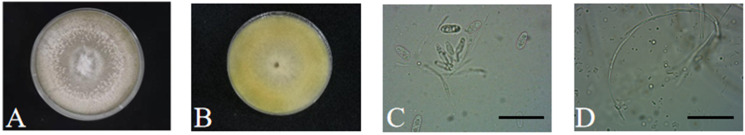
Colony morphology and spore morphology of the strain. (**A**) Front side of the pathogen after 5 days of growth. (**B**) Back side of the pathogen after 5 days of growth. (**C**) α-conidia (100×). (**D**) β-conidia (100×). Black bars represent 10 mm.

The pathogen B4 was isolated from passion fruit brown rot, using a tissue separation method (Figure 2). When strain B4 was grown on the PDA medium, the entire plate (90 mm) was covered within 5 days. The colonies were white, round, and concentric, the mycelium was plush, short, and flat, and the back of the medium was yellow. Microscopic analysis identified α-conidia and β-conidia in the B4 samples. The α-conidia were fusiform, single-celled, colorless, transparent, and without septa, but with an oil ball at each end. The β-conidia were filamentous, linear, colorless, transparent, and without septa.

### 3.3. Pathogenicity Testing of Pathogens

**Figure 3:**
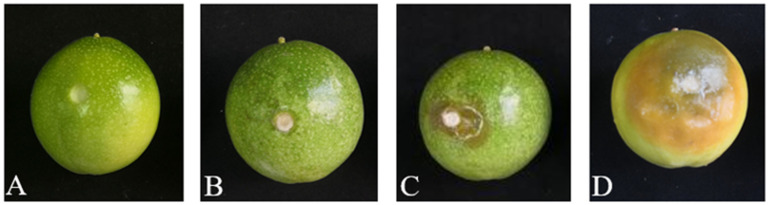
Pathogenicity test of isolated strains on passion fruit: (**A**) 0 d; (**B**) 1 d; (**C**) 3 d; (**D**) 5 d.

Strain B4 was inoculated on healthy passion fruit samples and its pathogenicity was assessed (Figure 3). One day after inoculation, white mycelium began to grow at the inoculation mouth (Figure 3B). Three days after inoculation, the fruit had watery brown spots, which are characteristic symptoms of brown rot (Figure 3C). Five days after inoculation, the symptoms of brown rot on the passion fruit had increased (Figure 3D). The initial brown spots expanded in diameter and eventually covered the entire fruit. The brown spots in the middle of the fruit deepened in color, while the water-stained edges continued to expand. The disease development eventually caused the fruits to rot completely. These observations indicated that strain B4 was highly pathogenic to passion fruit and could cause extensive rot.

### 3.4. Molecular Biology of the Pathogen

**Figure 4:**
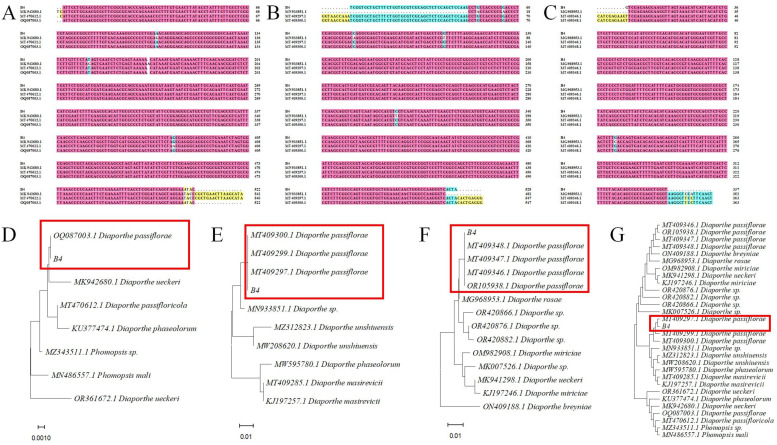
Molecular biological identification of isolated pathogen. (**A**) Sequence alignment based on rDNA-ITS sequence. (**B**) Sequence alignment based on TUB sequence. (**C**) Sequence alignment based on TEF-1α sequence. (**D**) Phylogenetic tree constructed for strain B4 based on rDNA-ITS sequence. (**E**) Phylogenetic tree constructed for strain B4 based on TUB sequences. (**F**) Phylogenetic tree constructed for strain B4 based on TEF-1α sequence. (**G**) Construction of a phylogenetic tree of strain B4 based on multiple sequences of rDNA-ITS, TUB, and TEF-1α. Branches including isolates obtained from the passion fruit are represented by red boxes.

According to the BLASTn analysis, strain B4 was 100% homologous to *D. passiflorae* (OQ087003.1) based on the ITS sequence and 100% homologous to *D. passiflorae* (MT409297.1, MT409300.1) based on the TUB sequence. Based on the TEF-1α sequence, strain B4 showed 100% homology with *D. passiflorae* (MT409348.1, MT409346.1). Sequence alignment based on the ITS, TUB, and TEF-1α genes displayed high similarity between strain B4 and *D. passiflorae* (Figure 4A–C). The phylogenetic analysis of the ITS sequences (Figure 4D) revealed that strain B4 clustered with *D. passiflorae* (OQ087003.1), while the phylogenetic analysis of the TUB sequences (Figure 4E) indicated that B4 was also clustered with *D. passiflorae* (MT409300.1, MT409299.1, MT409297.1). Similarly, the phylogenetic analysis of the TEF-1α sequences (Figure 4F) confirmed that B4 was clustered with *D. passiflorae* (MT409348.1, MT409347.1, MT409346.1, OR105938.1). The phylogenetic tree was constructed using neighbor joining on the combined dataset of the ITS, TUB, and TEF-1α sequences. According to Figure 4G, strain B4 and *D. passiflorae* with different accession numbers (MT409297.1, MT409300.1, MT409299.1) were clustered together in one branch, which indicates that strain B4 was most closely related to *D. passiflorae*. Therefore, combined with the pathogenicity test and morphological analysis, the pathogen B4 that was isolated from postharvest brown rot on passion fruit was identified as *D. passiflorae*.

### 3.5. Biological Characterization of D. passiflorae

**Figure 5:**
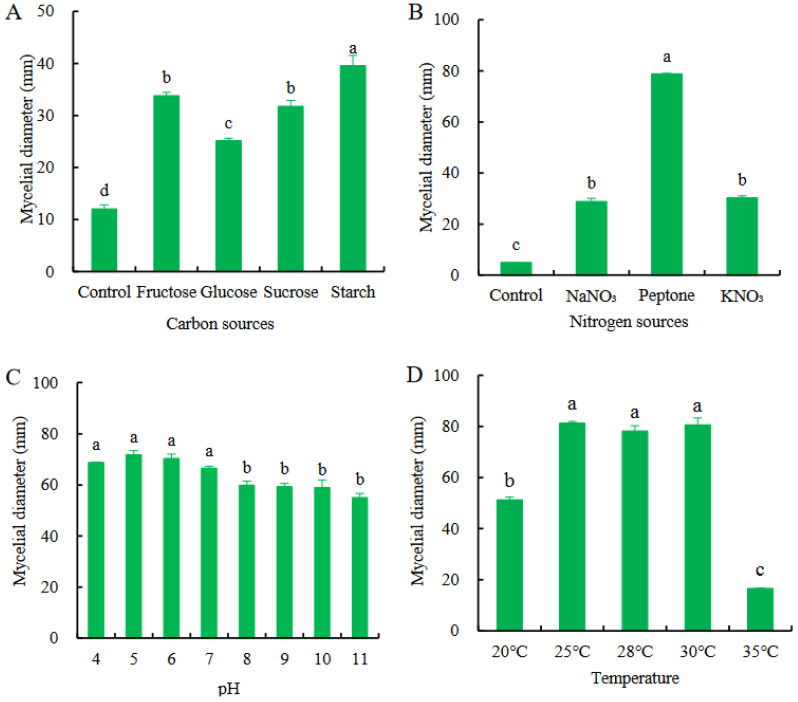
Biological characterization of *D. passiflorae*. (**A**) C-source. (**B**) N-source. (**C**) pH. (**D**) Temperature. The different letters above the columns indicate that the data were significantly different (*p* < 0.05) between groups.

*D. passiflorae* could grow in all four carbon sources tested and there were significant differences in the growth rates of the colonies (Figure 5A). The colonies grew relatively slowly on the control medium without sucrose and faster on the medium with the four carbon sources tested. Starch was used as the carbon source in the medium for the faster growth of the colonies. Mycelia developed, and the diameters of the colonies observed in the culture on day 4 were 39.56 ± 2.03 mm. Starch was determined to be the best carbon source.

*D. passiflorae* could grow on all three test nitrogen source media (Figure 5B). The colonies grew faster on the medium with peptone as the nitrogen source and the colony diameters were 78.83 ± 0.25 mm on day 4, which was highly significant (*p* < 0.01) when compared with the other experimental groups. The growth rate of the colonies was slowest on the base medium without sodium nitrate (control), as they were only 5 mm in diameter on day 4. These results show that peptone is the best nitrogen source for the growth of *D. passiflorae* colonies.

*D. passiflorae* could grow in a pH range of 4–11. At a pH of >8, the growth rate of the mycelia was slow, and the colony diameters were small and sparse. At pH 5, the colony growth rate was the fastest, and the colony diameters were 71.83 ± 1.59 mm. Compared with the other experimental groups with pH values > 8, the difference was significant (*p* < 0.05). At pH values of 5 and 6, there were no significant differences between the experimental groups (Figure 5C). These results showed that the growth of the passionflower colonies was suitable under acidic conditions, whereas it was inhibited under alkaline environments.

*D. passiflorae* grew at all five temperature gradients (Figure 5D). The colonies grew fastest at 25–30 °C, and the colony diameters were 78.26–81.3 mm after incubation for 4 days. However, the colony growth was slowest at 35 °C, and the colony diameters were 16.50 ± 0.24 mm after incubation for 4 days. These results showed that 25–30 °C was the optimum temperature range for the growth of *D. passiflorae*.

### 3.6. In Vivo Assay

#### 3.6.1. Effects of *D. passiflorae* on Spot Diameter

**Figure 6:**
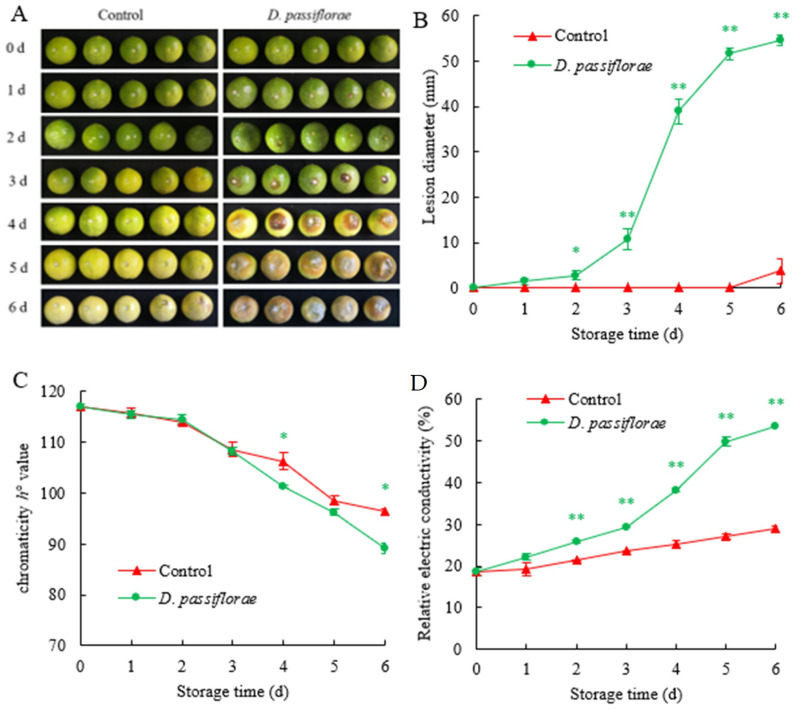
Effects of inoculation with *D. passiflorae* on fruit lesion diameter, *h°* value, and cell membrane permeability. (**A**) Symptoms of passion fruit brown rot. (**B**) Disease spot diameter. (**C**) *h°* value. (**D**) Cell membrane permeability. The marks * and ** represent the significant differences (*p* < 0.05 or *p* < 0.01) between the control group and the *D. passiflorae* inoculated passion fruits on each storage day. (▲) Control samples; (●) *D. passiflorae* samples.

The diameters of the brown rot spots on the passion fruits inoculated with *D. passiflorae* increased with the storage time (Figure 6A,B). Compared to the control, the diameters of the brown rot spots were significantly (*p* < 0.05) larger in the passion fruits inoculated with *D. passiflorae* at 2–6 d after harvest and reached a highly significant (*p* < 0.01) level at 3–6 d. The diameters of the diseased spots on the fruits inoculated with *D. passiflorae* rapidly increased at 2 d, while the fruit lesions in the control group started only at 6 d. Additionally, on day 6, the diameters of the passion fruit disease spots in the control group were 93% smaller than those in the inoculated *D. passiflorae* group.

#### 3.6.2. Effects of *D. passiflorae* Inoculation on the Fruit Hue Angle of Passion Fruit

The color change of the passion fruit was measured using the value of the hue angle. The postharvest passion fruit surface *h°* values continued to decline with the storage time, with the fruit gradually turning from green to yellow (Figure 6C). Compared to the control, the surface *h°* values of the passion fruits inoculated with *D. passiflorae* were lower at days 4–6 and the differences were significant (*p* < 0.05) on days 4 and 6. Additionally, when compared with day 0, the *h*° values of the fruits inoculated with *D. passiflorae* on day 6 were reduced by 24%.

#### 3.6.3. Effects of *D. passiflorae* Inoculation on Cell Membrane Permeability

The cell membrane permeability of the passion fruits increased with the storage time and showed a trend similar to that for the lesion diameter. Compared with the control group, the cell membrane permeability of the passion fruit inoculated with *D. passiflorae* was higher than that of the control group (Figure 6D). Additionally, it reached a highly significant level (*p* < 0.01) within 2–6 days. The cell membrane permeability of the control group was 29% on day 6, which was 46% lower than that with the *D. passiflorae* inoculation.

### 3.7. Effects of ε-PL, SA, and MT on the Mycelial Growth of D. passiflorae In Vitro

**Figure 7:**
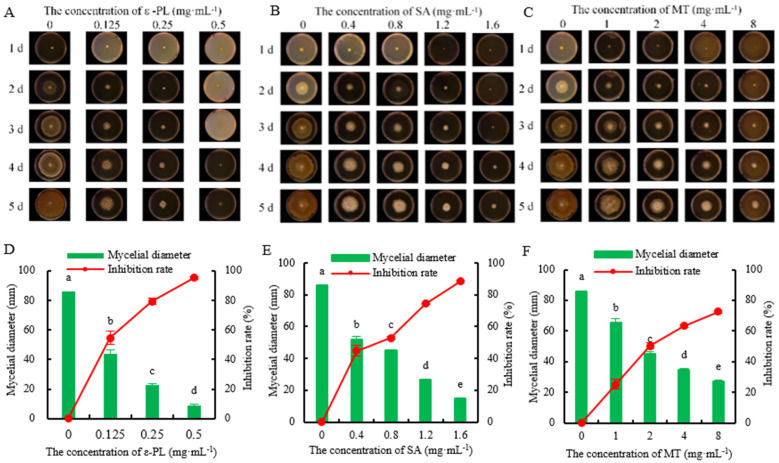
Effects of different concentrations of (**A**,**D**) ɛ-poly-l-lysine, (**B**,**E**) salicylic acid, and (**C**,**F**) melatonin on the mycelial growth and inhibition rate of *D. passiflorae* at 5 d in vitro. The different letters above the columns indicate that the data were significantly different (*p* < 0.05) between groups.

The ε-PL, SA, and MT treatments significantly (*p* < 0.05) inhibited the growth of the mycelia of *D. passiflorae*. As the concentrations of ε-PL, SA, and MT increased, the mycelial growth rate decreased continuously, and their inhibitory effects increased (Figure 7). The control group was completely overgrown on day 5, while the groups treated with the ε-PL, SA, and MT were not. The inhibition rates of 0.125, 0.25, and 0.50 mg/mL of ε-PL on the *D. passiflorae* were 54%, 79%, and 95%, respectively (Figure 7D). The inhibition rates of 0.4, 0.8, 1.2, and 1.6 mg/mL of SA on the *D. passiflorae* were 45%, 53%, 75%, and 88%, respectively (Figure 7E). The inhibition rates of 1, 2, 4, and 8 mg/mL of MT on the *D. passiflorae* were 29%, 53%, 65%, and 74%, respectively (Figure 7F). Therefore, ε-PL, SA, and MT all inhibited the growth of *D. passiflorae*, and ε-PL exhibited the best inhibitory effect (Table 1).

## 4. Discussion

Passion fruit is a tropical fruit that is highly sought after by consumers owing to its high nutritional and medicinal values [7,9,41], and it is consequently widely cultivated in China. However, because of the susceptibility of passion fruit to various diseases, it has a short shelf life, which negatively impacts its commercial value. Based on the treatment method used and environmental conditions, microbial infections may occur after harvest, resulting in postharvest rot [42]. Pathogens can enter from various parts of the fruit, such as mechanically damaged wounds or the stomata. In the early stages of disease, spots appear only on the peel; however, with the passage of time, the pathogen spreads in the fruit and then gradually into the pulp, resulting in fruit rot. During the early stages of brown rot in passion fruit, the only symptom is small brown lesions that appear on the peel (Figure 1). However, the diameters of these lesions gradually increase over time and, finally, the whole fruit decays completely. *Diaporthe infercuda* [16], *Phytophthora drechsleri* [17], *Phytophthora nicotianae* [18], *Colletotrichum brevisporum* [19], *Lasiodiplodia theobromae* [20], and *Trichothecium roseum* [21] have previously been reported as the causal agents of postharvest rot in passion fruit. However, in this study, *D. passiflorae* was isolated as the dominant pathogen causing brown rot in passion fruit. Microscope analysis showed that the B4 colonies were white and fluffy with short mycelia, and further evaluations revealed that they had α-conidia and β-conidia (Figure 2). The morphological analysis showed that the conidia of strain B4 were similar to those previously reported for *Diaporthe* [43]. The fruit pathogenicity of B4 was further tested, and the results demonstrated that the fruits inoculated with the B4 strain all showed symptoms of disease consistent with those of brown rot under natural conditions in passion fruit (Figure 3). Additionally, the rDNA-ITS gene sequences obtained via PCR amplification were analyzed for homology and a phylogenetic tree was constructed. Finally, combining the results of the morphological analysis, pathogenicity tests, and molecular identification, the dominant strain B4 was identified as *D. passiflorae*. Notably, *D. passiflorae* has previously been isolated from kiwifruit [44], but its role in causing postharvest decay in passion fruit has not been previously reported.

The effects of environmental factors on the growth of the fungal pathogen have been studied through its biological characteristics. Fu et al. [45] found that the optimal temperature range for *Phomopsis mangiferae Ahmad* growth was 25–30 °C. Yan et al. [46] found that the optimum carbon source for *Diaporthe eres* growth was starch and the optimum nitrogen source was peptone. In a recent study, Ariyawansa et al. [47] identified six *Diaporthe* species associated with leaf spot disease of *C. sinensis* in Taiwan tea fields, and found that the optimal temperature for mycelium growth was 25–30 °C, with a preference for growth in a low-acid–alkaline medium. In this study, through experiments employing different carbon and nitrogen sources, acid and alkaline conditions, and temperatures, the preference and growth requirements of the *D. passiflorae* strain to specific environments were analyzed. The results showed that *D. passiflorae* could thrive in an acidic environment at room temperature. We found that starch is the most suitable carbon source for its growth, while peptone is the preferred nitrogen source. The observations in this study are consistent with those of Fu et al. [45], Yan et al. [46], and Ariyawansa et al. [47].

Infection with pathogens can cause fruit diseases and rot [48]. Gong et al. [49] and Sun et al. [50] found that the fruit lesion diameter and cell membrane permeability increased after fungal infection. In the study, the lesion diameter (Figure 6B) and the cell membrane permeability (Figure 6D) of fruit inoculated with *D. passiflorae* increased and the decay symptoms were more pronounced, while the chromaticity *h°* values decreased (Figure 6C). Fruits uninoculated with *D. passiflorae* were not diseased on day 5 and maintained good-quality characteristics (Figure 6).

The development of the fruit industry has been seriously restricted by reductions in the commercial value of fruit due to microbial infection. Chemical fungicides have thus been widely used in fruit preservation to help maintain fruit quality and extend storage periods [24]. However, the long-term, irrational use of chemical fungicides can cause irreversible damage to human health and the environment [51]. Therefore, it is essential that we identify safe and effective treatment methods to inhibit the growth of pathogens and maintain fruit quality [52]. As a safe and nontoxic antifungal agent, ε-PL is widely used in food processing [53]. In recent years, it has been shown that ε-PL treatments can effectively inhibit the occurrence of postharvest disease in fruit. For example, the ε-PL treatment could effectively inhibit the occurrence of postharvest blue mold disease in apple, and it had an inhibitory effect on *Penicillium expansum* in vitro [26]. Additionally, ε-PL was found to have significant antifungal activity in vitro and could significantly inhibit the colony growth and spore germination of *Alternaria alternata* [54]. Similarly, SA and MT treatments could inhibit pathogen infection and thus maintain high fruit quality. SA treatments could reduce pathogen infections in mandarin fruit during cold storage and thus help maintain higher fruit quality [55]. It was found that the SA treatment could result in the protein leakage and lipid damage of the pathogen, thereby inhibiting the activity of *P. expansum* in vitro [56]. The occurrence of litchi downy blight was reduced and the resistance of litchi fruit was improved via the MT soaking treatment after harvest [57]. Meanwhile, it was found that the MT treatment of tomato fruits could suppress gray mold caused by *Botrytis cinerea* [58].

In the present work, the inhibitory effects of ε-PL, SA, and MT on *D. passiflorae* were studied. The results showed that 0.125 mg/mL ε-PL, 0.4 mg/mL SA, and 1 mg/mL MT could effectively inhibit the growth of *D. passiflorae* in vitro, and the inhibitory effect enhances with the increase in concentrations.

## 5. Conclusions

In summary, this study showed that the brown rot of passion fruit is caused by *D. passiflorae*. The infection with this pathogen causes significant postharvest disease. Furthermore, the research has clarified that the growth conditions of the fungal pathogen were determined by the biological characteristics of *D. passiflorae*. Moreover, the ε-PL, SA, and MT treatments inhibited the growth of *D. passiflorae* in vitro, among which the 0.5 mg/mL ε-PL treatment had the best inhibition effect. These findings not only improve our understanding of *D. passiflorae*, the passion fruit brown rot pathogen, but also provide valuable information for effective disease management.

## Figures and Tables

**Table 1:** Inhibition effects of ɛ-poly-l-lysine (ε-PL), salicylic acid (SA), and melatonin (MT) on *D. passiflorae*.

	The Concentration of ε-PL (mg·mL^−1^)				The Concentration of SA (mg·mL^−1^)				The Concentration of MT (mg·mL^−1^)			
	0	0.125	0.25	0.5	0.4	0.8	1.2	1.6	1	2	4	8
Diameter (mm)	86.00 ± 0.00 ^a^	43.68 ± 2.71 ^b^	22.54 ± 2.21 ^c^	9.13 ± 0.70 ^d^	52.08 ± 2.02 ^b^	44.98 ± 0.40 ^c^	26.66 ± 0.08 ^d^	14.90 ± 0.46 ^e^	65.59 ± 6.17 ^b^	45.19 ± 1.22 ^c^	34.77 ± 0.64 ^d^	27.22 ± 0.56 ^e^
Inhibition rate (%)	0 ± 0.00	52.25 ± 4.73	78.35 ± 2.10	94.90 ± 1.22	44.62 ± 3.36	52.96 ± 0.66	74.51 ± 0.14	88.35 ± 0.76	25.20 ± 3.28	50.26 ± 1.88	63.25 ± 0.96	72.57 ± 0.84

Different letters indicate significant differences (*p* < 0.05) between different concentrations in the same group.

## Data Availability

The sequences have been deposited in GenBank (Figure 4). The data presented in this study are publicly available at NCBI. Publicly available datasets were analyzed in this study. These data can be found here: https://www.ncbi.nlm.nih.gov/.
